# Robotics in Orthopaedics: Exploring Future Possibilities for South Africa

**DOI:** 10.7759/cureus.88998

**Published:** 2025-07-29

**Authors:** Wilhelm Hansen, Kofi Nyarko

**Affiliations:** 1 Department of General Surgery, University of the Witwatersrand, Johannesburg, ZAF; 2 Department of General Surgery, Chris Hani Baragwanath Academic Hospital, Johannesburg, ZAF

**Keywords:** cost-effectiveness, healthcare technology, orthopaedics, resource-limited settings, robotic-assisted surgery, south africa, surgical innovation, surgical training

## Abstract

This editorial examines the feasibility and ethical implications of integrating robotic-assisted orthopaedic surgery into South Africa’s healthcare system. While such technologies are well-established in high-income settings, enhancing surgical precision, reproducibility and postoperative outcomes, their widespread adoption in resource-limited contexts poses considerable logistical, financial and ethical challenges. The South African health landscape, marked by stark disparities between public and private sectors, provides a unique setting in which to assess whether such innovations should be prioritised over more pressing service delivery needs.

The article traces the evolution of surgical robotics and evaluates their role in joint arthroplasty and spinal instrumentation, noting that benefits often rely on specialised training, robust IT infrastructure and high patient volumes, conditions not uniformly met across public hospitals. The editorial proposes a phased, context-sensitive strategy: concentrate robotic capabilities in high-volume public centres, invest in simulation-based training and strengthen digital infrastructure through open standards and data governance frameworks.

Training is identified as a critical bottleneck in expanding access to robotic surgery. The piece advocates for national investment in simulation-based training using virtual and augmented reality (AR) platforms to expand skill development in a cost-effective and scalable way. These efforts could reduce dependency on international fellowships and enable the creation of a locally trained workforce. Additionally, successful adoption requires major upgrades in hospital IT systems, interoperability standards and data governance, without which robotic systems risk becoming underutilised and disconnected from broader clinical processes.

Ethical principles, such as justice, beneficence, non-maleficence and autonomy, anchor the discussion, guiding recommendations to ensure innovation supports rather than undermining equitable care. The editorial underscores the importance of aligning new technologies with principles of fairness, transparency and sustainability. It cautions against pursuing innovation for its own sake and argues for a balanced approach that ensures technological advancements do not exacerbate existing inequities in care. Alternatives such as image-guided navigation, AR and artificial intelligence-supported planning are explored as more scalable options for resource-constrained settings. A phased and context-sensitive model, emphasising modular technologies, planning tools and digital navigation systems, is recommended as a more viable entry point for surgical digitisation in South Africa.

Ultimately, the editorial advocates for a balanced approach, where surgical innovation is aligned with national priorities and serves the broader public good. While robotic orthopaedic surgery remains aspirational for much of South Africa’s healthcare system, its potential is not out of reach. With strategic planning, investment in human capital and attention to ethical imperatives, robotics can form part of a broader vision for advancing surgical care-one that integrates innovation with access, and precision with public value.

## Editorial

Robotic-assisted surgery has become a key aspect of precision medicine in orthopaedics, transforming practices in high-income countries through improved accuracy, consistency and clinical results in procedures such as total hip and knee replacements, spinal instrumentation and complex trauma repairs [1]. Beyond the operating room, these technologies are advancing areas like diagnostics, rehabilitation and patient monitoring [2], facilitated by the seamless integration of advanced hardware and software systems. However, as these innovations develop, important questions arise about their relevance and feasibility in low- and middle-income countries (LMICs), particularly in resource-constrained settings such as South Africa.

With a deeply divided healthcare system, limited access to basic surgical services and rising elective surgery backlogs, South Africa provides a unique context for evaluating the value, fairness and sustainability of costly surgical robotics [3]. This editorial challenges the idea that technological progress must follow the pattern established by wealthier nations and considers whether South Africa might instead develop a hybrid model, one that selectively adopts robotic capabilities suited to its specific healthcare challenges. For example, robotic-assisted laparoscopic prostatectomy and pedicle screw placement in complex spinal surgeries are candidates due to their demonstrated improvements in accuracy and reduced complication rates [1]. Selection criteria for adoption should include procedure complexity, institutional volume, existing perioperative infrastructure and the potential to offset long-term costs through reduced revision rates and shorter hospital stays [1]. Private sector involvement, such as through public-private partnerships, could also play a role in skill development, technology sharing and expanding access to training infrastructure [3].

Ultimately, the article assesses the practicalities and ethical considerations of implementing robotic-assisted surgery, where innovation must be carefully balanced with accessibility and systemic capacity. Surgical robotics, particularly in orthopaedics, marks a significant technological and cultural shift, improving surgical precision, consistency and planning through computer-guided systems, real-time data feedback and advanced tools such as haptic sensors and machine learning algorithms [1]. Systems like MAKO (Stryker, Mahwah, NJ) and Robotic Surgical Assistant (ROSA, Zimmer Biomet, Warsaw, IN) exemplify this progress, delivering submillimetre accuracy in joint replacements and spinal procedures, particularly in anatomically complex cases [1]. These innovations not only enhance clinical capabilities but also signal a broader move towards digitisation, standardisation and automation in surgery [1,2]. Nevertheless, while these developments promise better outcomes in well-resourced environments, their effectiveness is highly dependent on context.

In South Africa, where public healthcare often faces inconsistent theatre turnover, limited patient optimisation and uneven postoperative care, such technologies may struggle to deliver their full benefits [3]. As the surgical field becomes increasingly reliant on data, South Africa must critically evaluate which aspects of robotic surgery are essential, scalable and sustainable within its particular constraints. The key question is not only whether robotic surgery can be performed but also whether it should be prioritised over more fundamental healthcare needs.

The evolution of surgical robotics is rooted not only in technological innovation but also in a broader narrative of labour and assistance. The word 'robot' originates from the Czech term robota, meaning 'labour' or 'servitude' [4]. This concept materialised in 1985 when the first medical robot, the Programmable Universal Machine for Assembly (PUMA 200), performed a neurosurgical biopsy on a patient [4]. Initially used in neurosurgery, the PUMA system was later adapted for urological procedures, paving the way for its wider application in various surgical fields. A significant milestone was reached in 1992 with the development of the Robodoc Surgical System (Integrated Surgical Systems, Inc., Davis, CA), an image-guided robotic platform designed for hip replacements [4].

A major step forward occurred in 2000 when the U.S. Food and Drug Administration approved the da Vinci Surgical System, sparking widespread adoption of robotic-assisted surgery across multiple disciplines [4]. Since then, orthopaedic robotics have continued to evolve with platforms like MAKO and ROSA, specifically designed for joint replacements such as hip and knee arthroplasty. These systems incorporate state-of-the-art technologies, including artificial intelligence (AI), haptic feedback, real-time imaging and semi-autonomous functions that assist in precise bone preparation and implant alignment [1]. However, the development of surgical robotics also highlights a global divide. High-income countries, driven by consistent investment, strong training infrastructure and competitive healthcare markets, have quickly integrated these technologies into everyday practice. Conversely, LMICs have often remained marginal to this wave of innovation, hampered by financial constraints, limited training, and weaker health systems. South Africa epitomises this contrast, being home to some centres of excellence with advanced robotic capabilities, yet facing systemic disparities that restrict widespread adoption [3,5].

This divergence is not merely technological but strategic. The history of surgical robotics, from PUMA and Robodoc to da Vinci and beyond, provides lessons about engineering progress and the importance of contextual readiness and systemic integration [4]. In countries like South Africa, where healthcare resources are unevenly distributed and basic surgical services remain inaccessible to many, adopting high-cost technologies without a coordinated, evaluative approach risks exacerbating existing inequalities. Ultimately, the future of surgical robotics in such settings depends not only on the availability of technology but also on whether it can be integrated within a sustainable and equitable healthcare framework [5].

The clinical benefits of robotic-assisted orthopaedic surgery are well-supported by evidence showing improved implant alignment, fewer revision surgeries, shorter hospital stays and better postoperative recovery [1]. In spinal procedures, robotic guidance has also been demonstrated to improve the accuracy of pedicle screw placement, reducing neurological risks and enhancing fusion rates [1]. Nonetheless, these benefits are highly dependent on context and involve substantial financial and logistical challenges [3,5].

In South Africa, the costs of acquiring, maintaining and operating robotic systems, along with the extensive training requirements, raise important questions about sustainability and value [3]. These technologies demand a steep learning curve, with some studies suggesting that 100-200 cases are necessary to achieve consistent outcome improvements [5]. For low-volume centres, such investments may not be justified, especially when basic surgical services remain underfunded. Moreover, in South Africa’s overstretched public sector, where theatre delays, overcrowded wards and inadequate rehabilitation services persist, the theoretical benefits of robotics may not translate into real improvements [5].

Robotic surgical systems rely on real-time patient data, surgeon input and closed-loop feedback mechanisms to perform precise intraoperative actions [2]. These systems require high-speed, low-latency communication and must meet strict safety and performance standards, often achieved through modular, layered software architectures supported by real-time operating systems [2]. However, integrating these advanced platforms into hospital environments remains challenging, particularly in countries like South Africa, where many public hospitals still use outdated or paper-based systems [2]. A major obstacle is the absence of standardised communication protocols, such as Health Level Seven or Fast Healthcare Interoperability Resources, which hinders interoperability with core hospital systems, including Electronic Health Records and Picture Archiving and Communication Systems [2]. This lack of compatibility has already led to vendor lock-in and underutilisation of expensive robotic platforms in other LMICs, including Kenya and Nigeria [2]. To avoid similar issues, South Africa could build on open-source frameworks, such as Open Medical Record System and District Health Information Software 2, as a foundation for broader digital integration [2].

Additionally, cybersecurity is a growing concern. Robotic platforms handle sensitive patient data and are vulnerable to cyberattacks, necessitating strict adherence to international standards, such as the Health Insurance Portability and Accountability Act and the General Data Protection Regulation [2]. Without adequate data governance, upskilling of biomedical personnel and significant capital investment, South Africa’s public health sector may be ill-prepared to manage the complexities of integrating robotic surgery [2,5].

Since 2021, South Africa’s public health sector has acquired multiple da Vinci Xi Surgical Systems, each costing approximately R38 million. Installations at centres such as Groote Schuur Hospital and Tygerberg Hospital have facilitated over 500 procedures to date [5]. Implementation has included an extensive training infrastructure, simulation laboratories, wet laboratories, proctoring and multidisciplinary team involvement, marking a significant step in the country’s adoption of advanced surgical technologies [5]. However, this substantial investment has sparked ethical and economic debate. Critics, including Fagan, have highlighted the opportunity cost, noting that the R76 million spent on just two systems could have supported thousands of essential procedures, such as cataract surgeries, or addressed critical gaps in access to elective care [3].

With each robotic procedure costing approximately R65,000, driven by expenses related to single-use instruments, proprietary software and ongoing maintenance, the question of sustainability looms large in a fiscally constrained healthcare system [3]. Preliminary evaluations from local centres suggest intraoperative advantages, but formal cost-benefit analyses remain unpublished [5]. International data from LMICs reflect the varied economic viability of robotic surgery, which is often dependent on volume and infrastructure [2]. These may offer interim benchmarks for South Africa until local data becomes available. Moreover, concerns persist over whether high-tech interventions are appropriate in a system still grappling with basic surgical shortfalls and workforce limitations.

To reconcile innovation with equity, a more viable strategy may involve concentrating robotic platforms within high-volume, research-oriented centres where their capacity can be fully utilised and outcomes rigorously documented [5]. We propose the establishment of a national open-access robotic surgery outcomes registry, linked to accredited centres and academic partners. Standardised reporting of key metrics, such as complication rates, reoperations and functional outcomes, would enable iterative technology evaluation and inform evidence-based policy. Prospective studies and real-time data dashboards could further support evidence-informed scaling. Until then, large-scale expansion risks undermining broader healthcare objectives by diverting scarce resources away from more immediately impactful services.

In addition to robotics, South Africa could explore cost-effective alternatives, such as computer-assisted orthopaedic surgery, image-guided navigation, AI-supported decision tools and enhanced minimally invasive techniques. For example, navigation-assisted total knee arthroplasty has demonstrated comparable alignment accuracy to robotic systems at significantly lower cost, with reduced training requirements [1]. Augmented reality systems have also shown promise in improving surgical workflow in resource-constrained environments. These alternatives are particularly promising in low-resource settings with moderate surgical volumes [1].

South Africa has a rich legacy of surgical innovation, having pioneered minimally invasive surgery on the continent with the first laparoscopic cholecystectomy at Groote Schuur Hospital in 1990 [5]. The country later published groundbreaking work on laparoscopic radical cystoprostatectomy in 2018. Robotic surgery, with its enhanced precision and dexterity, especially in complex or anatomically challenging procedures, represents the logical next step in this trajectory [5]. However, unlike earlier advancements in minimally invasive surgery, robotic platforms demand significant infrastructure, sustained financial investment and partnerships with industry, factors that pose unique challenges within South Africa’s resource-constrained public healthcare sector.

While private institutions may adopt robotic surgery as a competitive differentiator, public hospitals must weigh its implementation against the broader mandate of equitable care [3]. Without a coordinated national framework to govern procurement, training and evaluation, the risk remains that robotics could deepen existing disparities rather than address them [3]. Instead of becoming a symbol of excellence accessible only to a privileged few, robotic surgery programmes should function as strategic test beds, generating data, refining protocols and informing scalable, resource-sensitive models that can benefit the wider system.

A national strategy for robotic surgery in South Africa could be built around three pillars: 1) centralised procurement to reduce acquisition costs, 2) accreditation of simulation-based training centres to standardise workforce preparation and 3) mandatory outcomes reporting tied to funding eligibility [3]. A dedicated electronic health (eHealth) task force, with representation from surgical societies, academic partners and government, could oversee implementation and ensure accountability. This strategy could be further supported by innovative financing mechanisms, such as lease-to-own procurement models, pooled purchasing agreements or public-private innovation funds [2].

The ethical implications of introducing high-cost robotic systems in a country like South Africa, where many still lack access to basic surgical procedures, are profound. The Lancet Commission on Global Surgery has declared equitable access to surgical care a global health priority, rendering it ethically problematic to divert scarce resources to high-tech solutions while essential services remain out of reach for the majority of the population [3]. Yet, the outright rejection of surgical robotics risks discarding potential long-term benefits. Innovation and equity can coexist, provided the approach is deliberate and grounded in ethics.

A dual-track model may offer a balanced path forward: elite centres can act as innovation incubators, refining robotic workflows and generating data, while select technologies such as navigation tools or planning algorithms are adapted for broader, resource-sensitive use across the system. For this to be ethically viable, implementation must be transparent, evidence-based and accompanied by community consultation. Additionally, robotic surgery may alter the patient-provider dynamic by creating perceptions of 'elite care'. This could erode trust in traditional services unless mitigated through equitable patient selection, transparent communication and education campaigns contextualising robotics as part of system-wide quality improvement [3]. Acknowledging these perceptions in the consent process and ensuring shared decision-making will be critical to building public trust. Robotic surgery in South Africa should not be about prestige; it should be about purpose, guided by a commitment to equity, accountability and sustainable impact.

The global trajectory of surgical robotics points toward deeper integration with AI, predictive analytics and even remote operations [1]. For South Africa, however, adopting these technologies must be intentional, context-sensitive and aligned with national health priorities. Rather than emulating high-income models, a phased, modular strategy is needed. This strategy leverages existing strengths and prioritises scalable impact by focusing on cost-effective technologies that can be integrated into current systems without overextending limited resources. Selective integration of digital components, such as computer-guided navigation, machine learning for surgical planning and virtual reality-based training, can offer many of the benefits of robotics without the prohibitive cost of full-system adoption [2]. These technologies can enhance precision, training and planning while remaining financially and logistically feasible. Critical enablers will include upgrading hospital information technology infrastructure, adopting open data and communication standards, and ensuring system interoperability. International collaborations can support training and research, with innovations like tele-mentoring and real-time remote proctoring helping to bridge existing skills gaps [2,3].

Ultimately, South Africa’s path must be guided not only by technological possibilities but also by ethical oversight, robust data and inclusive governance. The goal should not be technological prestige but health equity, ensuring that innovation strengthens, rather than widens, existing healthcare disparities. While the editorial emphasises high-volume centres as initial test beds, it recognises the risk of reinforcing geographic disparities. A longer term vision must include gradual decentralisation, leveraging mobile units, remote mentoring and distributed simulation hubs to reach underserved areas.

Robotic-assisted orthopaedic surgery remains largely aspirational within South Africa’s healthcare system, but it holds significant transformative potential if pursued with strategic intent and equity at its core. Its success will depend not on technological inevitability but on deliberate choices grounded in data, infrastructure readiness and a commitment to broad-based impact. While preliminary evaluations from academic centres suggest improved intraoperative outcomes, these benefits remain largely speculative in the South African context due to the lack of robust local cost-effectiveness data. Comprehensive cost-benefit analyses have yet to be published. Benchmarking against countries such as Brazil and India, which share similar socioeconomic conditions, may provide useful guidance while local evidence is developed.

The integration of robotics should be seen not merely as a symbol of medical progress but as a component of a broader innovation agenda that enhances care without compromising access, quality or fiscal sustainability. Realising this vision will require multi-sector collaboration, robust policy frameworks and targeted investment in both digital and human infrastructure.

Crucially, robotic surgery in South Africa must align with the principles of bioethics. Justice demands that innovations benefit everyone, not just the privileged few, and that limited resources are distributed fairly and equitably. Non-maleficence obliges decision-makers to avoid harm, not only to patients but also to the broader system, by not displacing vital services for the sake of technological innovation. Beneficence urges us to pursue robotics only if it genuinely improves outcomes and system performance. Autonomy requires patients and practitioners to engage with robotic interventions through informed consent, supported by transparent data and options. Only by grounding innovation in these ethical principles can South Africa responsibly lead in equitable, technologically advanced surgical care.
